# Piezoelectric scattering limited mobility of hybrid organic-inorganic perovskites CH_3_NH_3_PbI_3_

**DOI:** 10.1038/srep41860

**Published:** 2017-02-02

**Authors:** Ying-Bo Lu, Xianghua Kong, Xiaobin Chen, David G. Cooke, Hong Guo

**Affiliations:** 1 School of Space Science and Physics, Shandong University, Weihai 264209, China; 2 Department of Physics, McGill University, Montreal, QC H3A 2T8, Canada; 3 School of Physics and Energy, Shenzhen University, Shenzhen 518060, China

## Abstract

Carrier mobility is one of the most important parameters for semiconducting materials and their use in optoelectronic devices. Here we report a systematic first principles analysis of the acoustic phonon scattering mechanism that limits the mobility of CH_3_NH_3_PbI_3_ (MAPbI_3_) perovskites. Due to the unique hybrid organic-inorganic structure, the mechanical, electronic and transport properties are dominated by the same factor, *i*.*e*. the weak interatomic bond and the easy rotation of methylammonium (MA) molecules under strain. Both factors make MAPbI_3_ soft. Rotation of MA molecule induces a transverse shift between Pb and I atoms, resulting in a very low deformation potential and a strong piezoelectricity in MAPbI_3_. Hence the carrier mobility of pristine MAPbI_3_ is limited by the piezoelectric scattering, which is consistent to the form of its temperature dependence. Our calculations suggest that in the pristine limit, a high mobility of about several thousand *cm*^2^ *V*^−1^ *S*^−1^ is expected for MAPbI_3_.

Hybrid organic-inorganic perovskites (HOIPs) have been known for a long time, but there has been a recent explosion of interest in these materials due to their exceptional performance as the active material in a solar cell. Within five years, the power conversion efficiency (PCE) of HOIPs solar cell has increased from 3.8% to 22.1%^1^,^2^,^3^,^4^,^5^. The excellent performance mainly originates from the hybrid organic-inorganic structure which possesses favorable characteristics such as high electrical mobility, band gap tunability, excellent optical absorption and low fabrication cost^6^. Listed among the several favourable properties amenable to optoelectronic devices, the excellent transport properties of HOIPs are partly responsible for the large PCEs, which combined with their long carrier lifetimes, governs the large diffusion lengths characteristic of these materials^7^,^8^. Charge carrier mobilities in high quality polycrystalline films are consistently found in the 10–60 *cm*^2^ *V*^−1^ *S*^−1^ range, determined by time-resolved THz spectroscopy (TRTS)^9^,^10^,^11^, time-resolved microwave^12^ and Hall measurements^13^,^14^. For large single crystal samples, Stoumpos *et al*. reported^15^ that MASnI_3_ possesses a mobility exceeding 2000 *cm*^2^ *V*^−1^ *s*^−1^; and more recently using ultra-broadband TRTS Cooke *et al*. measured a pure Drude response and a remarkably high transient mobility of 500–800 *cm*^2^ *V*^−1^ *s*^−1^ on picosecond time scales for methylammonium (MA) lead iodide (CH_3_NH_3_PbI_3_ or MAPbI_3_) materials^16^. These and some other reported mobilities of HOIPs are tabulated in Table S1 in the supplementary material. We note that these reported high mobilities are comparable to that of traditional semiconductors, *e*.*g*. GaN and AlN^17^.

The most intensively investigated HOIPs to date is MAPbI_3_, however the intrinsic limitations of electrical transport in this material are still under experimental and theoretical investigation. For most semiconductors, various scattering mechanisms between charge carriers and phonons or impurities limit their transport properties. For example, from the broadening of the photoluminescence linewidth of MAPbI_3_, a polar optical phonon limited carrier mobility was proposed^14^,^18^,^19^,^20^. However, since MAPbI_3_ is a light-sensitive material, several effects including photostriction^21^, degradation due to above-band-gap illumination^22^,^23^, band structure variation caused by spin-orbital coupling (SOC)^24^ etc., influence the measured photoluminescence lineshape. It is also difficult to separate competing contributions from acoustic phonons and optical phonons. For MAPbI_3_, several experiments reported^11^,^14^,^25^,^26^,^27^ a temperature dependence of the carrier mobility to be *μ* ∝ *T*^−1.5^, which could not be easily explained if the scattering mechanism is due solely to the polar optical phonon. On the other hand, such a temperature dependence suggested a scattering mechanism due to deformation potentials, *i*.*e*. one of the acoustic phonon scattering mechanisms, because a *T*^−1.5^ factor does appear in the mobility formula^28^,^29^,^30^,^31^,^32^. Nevertheless, in the same mobility formula, the carrier effective mass m* may also be temperature dependent, for instance m* ∝ *T*^0.4^ in lead chalcogenides^33^,^34^. Therefore if this temperature dependence were also true for MAPbI_3_, the mobility governed by deformation potential would become 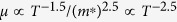, which is markedly different from the reported experimental data^11^,^14^,^25^,^26^,^27^ of *μ* ∝ T^−1.5^. Most importantly, in fact some smaller exponents were also observed experimentally^11^, *μ* ∝ [*T*^−1.2^, *T*^−1.4^]. Therefore it appears that the deformation potential scattering mechanism alone cannot explain the experimentally observed temperature dependence of MAPbI_3_ mobility. In fact, the mobility measured in optically pumped samples may be affected by electron-hole scattering to alter the temperature dependence^11^,^25^,^26^,^35^. With these and other complications, so far no clear consensus has emerged concerning the dominating scattering mechanism that limits the carrier mobility of pristine MAPbI_3_.

Interestingly and known experimentally, MAPbI_3_ with tetragonal symmetry (I4/mcm) is ferroelectric and piezoelectric, and a light-enhanced piezoelectricity has been observed^36^,^37^,^38^. Theoretically, Frost *et al*. predicted a large polarization value of 38 *μC*/*cm*^2^ for MAPbI_3_ materials^39^. The piezoelectric scattering - a type of the acoustic phonon scattering, is also suggested to play a dominant role for mobilities of certain semiconductors such as GaN with large, built-in internal electric fields^40^,^41^. An important and interesting question therefore arises: what is the role of piezoelectric scattering in hybrid organic-inorganic perovskites MAPbI_3_? It is the purpose of this work to investigate this issue theoretically.

We focus on elucidating the role of piezoelectric scattering to the carrier mobility of MAPbI_3_, using first principles techniques. The mechanical, electronic and transport properties of this material are found to be related and heavily influenced by the weak interatomic bonds and the easy rotation of MA molecules under strain. The acoustic phonons do not manifest deformation potential scattering, but bring about a strong polarized electric field which gives piezoelectric scattering as the dominant mobility limiting scattering mechanism in pristine MAPbI_3_. We also explain the observed temperature dependence of mobility through the piezoelectric scattering mechanism.

## Results

### Geometric structure of tetragonal MAPbI_3_

**Figure 1:**
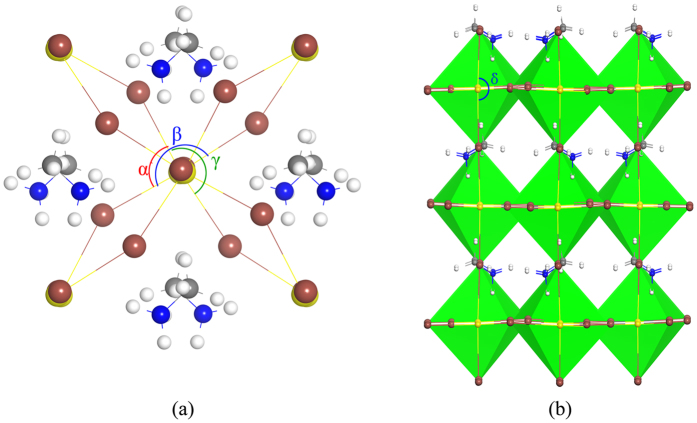
(**a**) Top view and (**b**) side view of the geometric structure of tetragonal MAPbI_3_. The yellow, brown, blue, grey and white balls represent Pb, I, N, C and H atoms, respectively. *α, β, γ* and *δ* in (**a**,**b**) denote angles of I-Pb-I, which show the distortion of PbI_6_ octahedra.

As illustrated in Fig. 1, the tetragonal MAPbI_3_ is constituted by networks of corner-sharing PbI_6_ octahedra, with organic MA molecules fitting into small cuboctahedral holes. MA molecules provide two important roles to MAPbI_3_: maintaining charge neutrality and stabilizing the perovskite structure^42^,^43^. Since the MA molecule is dipolar, the crystal structure and thus the electronic structure of MAPbI_3_ is extremely sensitive to the orientation of the MA molecule. We have investigated many MAPbI_3_ configurations with MA molecules arranged in different patterns (shown in Fig. S1 in supplementary materials) using density functional theory (DFT) total energy method^43^,^44^,^45^, and find that the most energetically stable structure is one where all the MA molecules are oriented along the [110] direction but arranged perpendicular to each other, as shown in Fig. 1. Because the bonding interactions at two radicals of MA molecules are different, obvious tilting of PbI_6_ octahedrons and out-of-plane rotations of MA molecules appear. We therefore use this configuration as the standard model structure of tetragonal MAPbI_3_ in the rest of this work unless explicitly specified.

### Electronic structure of tetragonal MAPbI_3_

Band structures and band gaps of MAPbI_3_ calculated by various strategies with DFT are shown in Fig. S2 and Table S2 in supplementary materials, respectively. The band gap calculated by DFT with the generalized gradient approximation (GGA) is 1.552 eV. The inclusion of HSE functional and/or spin-orbit coupling (SOC) interaction provides corresponding gap corrections, and the result agrees well with the experimental value^46^. Another key parameter with respect to transport is the effective mass of carriers (m*) that can be obtained by parabolic band fitting around the Γ point in the Brillouin zone^47^,^48^. The effective carrier mass tensors obtained by various approaches are listed in Table S2 in supplementary materials. The inclusion of SOC reduces m* significantly, especially for electrons, 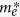 decreases from the DFT-GGA value of ~0.900 *m*_*e*_ to the DFT-SOC value of ~0.160 *m*_*e*_, where *m*_*e*_ is the bare electron mass. This is reasonable because SOC impacts only on heavy elements. The introduction of SOC in the calculation therefore strongly influences the dispersion near the conduction band minimum (CBM) of MAPbI_3_, which is governed by the Pb-6p orbitals^49^,^50^. Nevertheless, compared with DFT/DFT-SOC functionals, the more advanced HSE/HSE-SOC approaches do not introduce a sizeable change to the effective mass of both electrons and holes. Hereafter in the discussions below, we use the effective mass obtained from DFT-SOC since these values are close to the obtained experimental values^51^.

A knowledge of the carrier effective mass is necessary to estimate the charge carrier mobility, however more information is required. Next, we investigate how carriers interact with the host material when they traverse the lattice. In general, the intrinsic mobility is limited by a variety of scattering mechanisms, including impurity scattering, boundary scattering, phonon scattering, etc. Previous work has suggested impurity scattering plays less of a role in limiting the observed MAPbI_3_ mobility^6^,^11^,^44^,^45^,^52^. The experimentally observed temperature dependence of the mobility was predominantly attributed to acoustic phonon scattering^17^ which is composed of deformation potential scattering and piezoelectric scattering. In the following we investigate these scattering mechanisms individually.

### Deformation potential scattering

The acoustic phonons induce lattice vibration which results in the periodic shift of band edges. These potential shifts act as a source of carrier scattering, called deformation potential scattering. By Eq. 1 in the Method Section below, we calculate the mobility *μ*_*dp*_ of tetragonal MAPbI_3_ due to the deformation potential scattering mechanism where the temperature is set at 300 *K*. The results are listed in Table 1 where the mobility for electrons 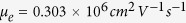, and for holes 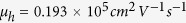. As tetragonal MAPbI_3_ is not an isotropic material, we calculate mobilities projected into three lattice axis using Eq. 2 in the Method Section and list them in Table 1. We find that the calculated *μ*_*dp*_ of MAPbI_3_ is extremely large, much larger than the highest experimental value of 800 *cm*^2^ *V*^−1^ *s*^−1^. This is caused by two factors: the small carrier effective mass m* and the low deformation potential Ξ of the material. Particularly, a low deformation potential means the scattering of acoustic phonons with carriers is rather weak, resulting in a relatively large mobility. We conclude that such a very weak deformation potential is likely not the limiting factor to the observed carrier mobilities of MAPbI_3_.

### Piezoelectric scattering

Acoustic phonon scattering is usually described by the deformation potential which, as we showed above, does not appear to provide the limiting scattering mechanism for the mobility of MAPbI_3_. However for certain semiconductors, namely for non-centrosymmetric crystals, the elastic strain can create macroscopic electric fields that induce a coupling between acoustic waves and free carriers, known as piezoelectric scattering. In semiconductors with high piezoelectric coefficient such as GaN, ZnO etc., piezoelectric scattering actually dominates over the deformation potential scattering^40^,^41^. The tetragonal MAPbI_3_ belongs to the space group I4/mcm and has a strong piezoelectric response^38^,^39^,^53^. Normally the piezoelectric strain coefficient *d*_*zz*_ of MAPbI_3_ is ~5–6 pm/V, but the light-enhanced piezoelectric coefficient dramatically increases up to 25 pm/V^36,37^. Therefore the contribution of piezoelectric scattering to carrier mobility in MAPbI_3_ cannot be ignored.

Table 2 summarizes the piezoelectric stress coefficients *e*_15_, *e*_31_ and *e*_33_ obtained by our first principles calculation, together with the elastic constant tensor adopted from experiments^54^. Putting these parameters into Eq. 3 in the Method Section, we obtain the piezoelectric scattering limited mobility *μ*_*pe*_, as listed in Table 2. *μ*_*pe*_ for electrons and holes are found to be 11294 cm^2^ *V*^−1^ *s*^−1^ and 8368 *cm*^2^ *V*^−1^ *s*^−1^, respectively. The piezoelectric scattering limited mobility *μ*_*pe*_ shows two interesting characters. First, the hole mobility is smaller than that of the electron, due to the larger effective mass of the hole. Second, the mobility obtained from piezoelectric scattering is significantly lower than that obtained by the deformation potential scattering: for electron *μ*_*pe*_ < *μ*_*dp*_ dramatically by two orders of magnitude. We conclude that for carriers in MAPbI_3_, piezoelectric scattering dominates over the deformation potential scattering.

## Discussions

It is quite surprising that piezoelectric scattering, rather than deformation potential scattering, is a stronger influence on the mobility of MAPbI_3_. Note that the piezoelectric scattering mechanism for the hybrid organohalide Pb-based material has not been considered previously, and some discussions are in order. In particular there are two issues to address: why the deformation potential scattering gives a very high mobility in MAPbI_3_, and why piezoelectric scattering plays a critical role. While the small effective mass already points to a large mobility, the form of the deformation potential arising from the unique geometric structure of MAPbI_3_ is key to this result and is discussed below.

All existing experimental and theoretical investigations have revealed the extremely soft nature of the HOIPs materials^54^,^55^,^56^. For example, MAPbI_3_ is known to have good ductility and its bulk modulus is only 10–20 GPa. The soft nature is attributed to two reasons: the weak bonding interaction and the easy rotation of the MA molecules under strain. For the optimized MAPbI_3_ structure obtained in our calculation, the Pb-I bond lengths, the C-I bond lengths and N-I bond lengths are found to be 3.14–3.22 *Å*, 3.84–4.00 *Å*, and 3.61–3.84 *Å*, respectively. These values are much longer than the bond lengths of traditional semiconductors such as Si, Ge and In_2_O_3_, etc.^57^. The longer bond length implies a relatively weak interatomic bonds^58^ and thus weak resistance to elastic deformation. Therefore a small elastic constant and a soft character of the MAPbI_3_ material are expected.

On the other hand, the influence on mechanical properties by rotations of the MA molecule is seldom considered, even though it is discussed in studies of other physical properties. The weak interatomic bonds between Pb-I and the hydrogen bonding between MA molecules and inorganic units, give rise to a very low rotational barrier for the MA molecules under external strain and/or intrinsic lattice disturbances. The calculated rotation angles of MA molecules are tabulated in Table S3 in supplementary materials, where *θ*_*z*_, *θ*_*y*_ represent angles between the C-N bond direction and the *xy*-plane or *xz*-plane, respectively. Variations of *θ*_*z*_ and *θ*_*y*_ show that when the strain in the *x*-axis changes from tension to compression, the C-N bond direction (*i*.*e*., the direction of the MA molecule) tends to rotate toward the *z*-axis and *y*-axis, respectively. Furthermore, if the strain is along *y*-axis and *z*-axis, the MA molecules also rotate towards the *xz*- and *xy*-planes, respectively. These findings mean that if the MAPbI_3_ lattice is subjected to a strain along one specific direction, the MA molecules will rotate toward other two perpendicular directions, which releases resistance to the external strain. Therefore, for a given strain, with MA molecular rotation the MAPbI_3_ lattice changes more significantly than without the rotation, contributing further to a low elastic constant and soft nature of the material.

**Figure 2:**
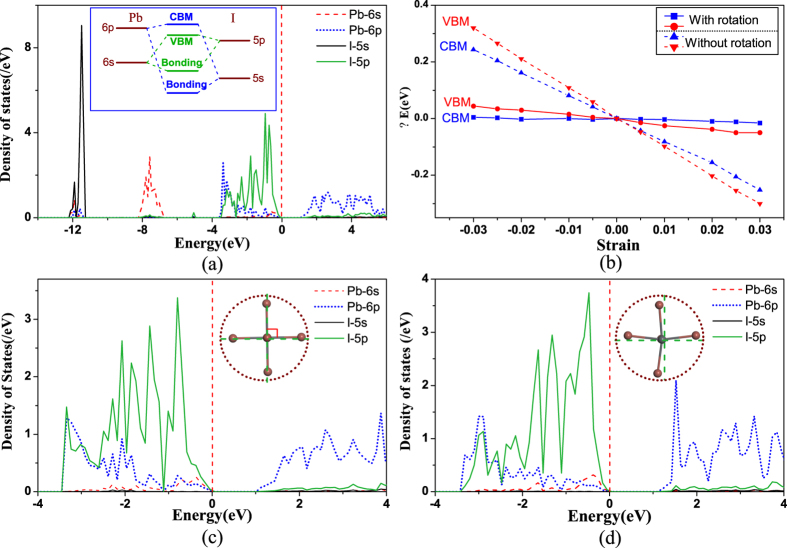
(**a**) PDOS of MAPbI_3_ materials and the inset represents the schematic diagram for the coupling interaction near band edges. (**b**) Energy shifts of VBM and CBM caused by the external strain with the presence and absence of rotation of MA molecule, respectively. (**c**,**d**) Are PDOS of MAPbI_3_ without and with the distortion of PbI_6_ octahedra, respectively. Insets in (**c**,**d**) illustrate the standard PbI_6_ octahedron and tilting PbI_6_ octahedron, respectively.

Coincidentally, the combination of weak interatomic bonds and rotations of the MA molecules in MAPbI_3_ are the exact reason why MAPbI_3_ possesses a rather low deformation potential. The conduction band minimum (CBM) of MAPbI_3_ is the anti-bonding state formed by the Pb-6p and I-5s orbitals, while the valence band maximum (VBM) is the anti-bonding states composed of the Pb-6s and I-5p orbitals, as illustrated in Fig. 2(a). Generally speaking, if the volumetric variation only leads to compression (dilation) of the bond length, both CBM and VBM of MAPbI_3_ would shift upward (downward), due to the enhanced (weakened) repulsion between the s and p orbitals, as plotted in Fig. 2(b). The deformation potential is inversely proportional to the energy difference between the two constituent atomic orbitals making up the anti-bonding states^59^. A higher deformation potential of the VBM than that of the CBM is obtained due to the relatively smaller energy difference between I-5p and Pb-6s compared to that of I-5s and Pb-6p.

Besides the change of bond length, lattice strain changes the inorganic PbI networks to adapt to the rotation of the MA molecules. We use the I-Pb-I angle to illustrate the distortion of PbI_6_ octahedra, where *α, β, γ* in Fig. 1(a) represent the in-plane tilting angles, and *δ* in Fig. 1(b) denotes the out-of-plane tilting angle. Data in Table S3 in supplementary materials show that with the increase of strain (from compression to tension), all angles *α, β, γ* and *δ* decrease, indicating the distortion of PbI_6_ octahedron is reinforced. It also means that the relative transverse shift between Pb and I atoms inside the PbI_6_ octahedron is enlarged. In ref. 60, Kim *et al*. demonstrated that the relative transverse displacement between Pb and I atoms raises the band edge levels, because the coupling between Pb-6p and I-5p changes from non-bonding states to anti-bonding states. Figure 2(c,d) illustrate the projected density of states (PDOS) of MAPbI_3_ before and after the transverse atomic displacement. We can clearly find that after the atomic shift, both intensities of I-5p states in CBM and Pb-6p states in VBM increase, indicating that the anti-bonding interactions are strengthened for both CBM and VBM. Hence, as aforementioned, the increase of strain (volumetric expansion) leads to an increase of Pb-I bond length as well as a transverse shift between Pb and I atoms. The former factor shifts both CBM and VBM downward, while the latter raises them. In some sense, these two conflicting factors “cancel” as far as the variation of CBM and VBM is concerned, namely their deformation potentials take small values.

To further examine effects induced by distortion of PbI_6_ octahedron and transverse shift between Pb and I atoms, we adopt another approach where we only change the Pb-I bond length while preventing the MA molecules from rotating and the transverse shift between Pb and I atoms to change. The calculated deformation potentials of MAPbI_3_ by this approach are also illustrated in Fig. 2(b) which are around 8 eV and 10 eV for CBM and VBM, respectively. The deformation potentials without the atomic transverse shift are much larger than the value with atomic shift, revealing that the relatively transverse shift between Pb and I atoms is one of the key reasons to reduce the deformation potential in MAPbI_3_. Nevertheless, we should note that in comparison with other materials such as GaN and PbS, the deformation potentials of MAPbI_3_ calculated without atomic shift still take lower values even though they are 8.0–10.0 eV^33^, arising from the weak interatomic bonding interaction. We conclude that the combined effect of the weak bonding interaction and the atomic transverse shift inside PbI_6_ octahedra, produces a low deformation potential. As a result, the deformation potential scattering mechanism plays only a minor role to the carrier mobility of MAPbI_3_.

As discussed above, MAPbI_3_ exhibits pronounced piezoelectricity, due to a weak bonding interaction and rotation of the MA molecules. The MA molecule has a permanent dipole due to the imbalance of charge distribution between the methyl radical and the ammonia radical, and the piezoelectric effect of MAPbI_3_ has been attributed to the dipole variation^36^. Note that the role of the MA molecule includes not only its own dipole, but also the transverse shift between the Pb and I atoms. The latter enhances the separation between the positive charge center (*i*.*e*., Pb atom) and the negative charge center (*i*.*e*., I atom) inside the PbI_6_ octahedra. This provides another contribution to the piezoelectric response of MAPbI_3_. Our calculated Born effective charges of Pb, I, C and N atoms are 4.693 *e*, −2.624 *e*, 0.314 *e*, and −0.620 *e*, respectively. The latter two values produce a dipole on the MA molecule roughly equals ~2.250 Debye. When the strain upon *z*-axis changes from −0.030 to +0.030, the average change of separations between the Pb and I atoms is nearly 0.04 *Å*, hence the distortion of PbI_6_ octahedron gives a change of polarization of about 1.246 *μC*/*cm*^2^. The detailed piezoelectric stress coefficients are given in Table 2. The piezoelectric response in MAPbI_3_ means that a polarization field can be induced by lattice strain: the strain can be applied externally or induced internally by acoustic phonons. Carriers then interact with the polarization field and are scattered during the transport process. The strong piezoelectricity of MAPbI_3_ gives rise to pronounced piezoelectric scattering, thus a much lower carrier mobility than that produced by the deformation potential scattering.

The temperature dependence of mobility can be used as an indicator for elucidating the scattering mechanism. For the mobility of MAPbI_3_, several experimental studies reported^11^,^25^,^26^,^27^ a temperature dependence of *μ* ∝ *T*^−1.2^ − *T*^−1.6^. La-o-vorakiat *et al*.^19^ reported that the carrier relaxation time follows a power law of *τ* ∝ *T*^−0.42^. If the effective mass m* were temperature independent, the deformation potential scattering would indeed give *μ* ∝ *T*^−1.5^ through Eq. 1 - in the range of the reported experimental temperature power of 1.2–1.6. However, for many semiconductors, the nonparabolicity effect in the band edge and thermal expansion in the material cause m* to vary significantly with temperature. For instance, m* ∝ *T*^0.4^ has been well-known for Si, PbSe and PbS^34^,^61^,^62^. Hence for these materials, the mobility governed by deformation potential scattering is *μ* ∝ 1/[(m*)^2.5^ *T*^1.5^]∝*T*^−2.5^ (see Eq. 1), in excellent consistency with the experimentally measured power law of *μ* ∝ *T*^−2.20^ for Si^63^; *μ* ∝ *T*^−2.55^ for PbSe^33^,^34^; and 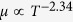 for PbS. Therefore, if MAPbI_3_ were to have a temperature dependence of its effective mass, the deformation potential scattering would not necessarily yield a power law of 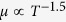.

Next, let’s consider piezoelectric scattering where the temperature dependence of the mobility is given by Eq. 3, *i*.*e*. 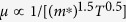. For MAPbI_3_, though there is still no experimentally reported temperature dependence of m*, we may employ 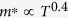 - known for lead dihalides^17^ which have similar mechanical properties as MAPbI_3_, and this gives a power law of 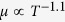. This exponent is rather close to the experimental reported 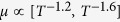 power law^11^. In addition, the carrier relaxation time deduced from piezoelectric scattering follows 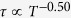, consistent with the experimentally observed^19^ 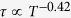. We conclude that the experimentally observed temperature dependence of the mobility of MAPbI_3_ appears to be most consistent with the piezoelectric scattering.

Note that the calculated mobility value as limited by the piezoelectric scattering for MAPbI_3_ is still about a factor of ten higher than the so far reported experimental values. This difference can be due to several reasons. First, a light-enhanced piezoelectricity of MAPbI_3_ was reported experimentally^36^ where a rapid increase of piezoelectric coefficient under illumination was detected, and this effect could increase the square of piezoelectric stress constant in Eq. 3 by a few times so that the mobility is reduced. Second, in real materials there can be scattering by grain boundaries (GB) to reduce the observed mobility^12^,^27^. GB scattering is very complicated but has been well studied in the semiconductor literature^64^. If the carriers are trapped by GBs, one expects an exponential temperature dependence of transport due to the activation energy. On the other hand, carriers can also quantum tunnel through the GBs^64^ which is a process independent of temperature, and GB tunneling will clearly reduce the measured mobility. In this scenario, the temperature dependence of the mobility would be that of the next dominant scattering mechanism, which our results suggest to be the piezoelectric scattering. Indeed, it has been reported experimentally that GB scattering plays an important role in MAPbI_3_ films^12^,^27^. Therefore, the calculated piezoelectric scattering limited carrier mobility of about 8000–10000 *cm*^2^ *V*^−1^ *s*^−1^ can be taken as the upper limit for pristine bulk MAPbI_3_.

### Summary

We have carried out systematic first principles calculations to determine the possible microscopic acoustic phonon scattering mechanism that limits the carrier mobility of perovskites MAPbI_3_. Our calculations reveal a very close relationship between mechanical, electronic and transport properties of these materials. The soft nature of MAPbI_3_ originates from the weak Pb-I bond/MA-I bond and the easy rotation of MA molecules, indicating that the structural fluctuations and dynamical disorder could emerge easily in MAPbI_3_. The weak Pb-I bond and the rotation of MA molecule also produce a transverse shift between Pb and I atoms inside PbI_6_ octahedron under lattice strain, which in turn raises the anti-bonding states near the band edge and reduces the deformation potential of MAPbI_3_ to a very low level.

On the other hand, the rotation of MA molecule changes its dipole. The transverse shift between Pb and I atoms separates the positive and negative charge centers. Both factors give rise to the strong piezoelectricity of MAPbI_3_. Therefore, our calculations suggest that the main effect of acoustic phonons in MAPbI_3_ is to generate a strong piezoelectric scattering. By considering the temperature dependence of the effective mass, we argue that the temperature dependence of mobility - as limited by the piezoelectric scattering, is 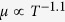, which is close to the reported experimental power law of *T*^−1.2^ to *T*^−1.6^. The calculated piezoelectric scattering limited mobility of about 8000–10000 *cm*^2^ *V*^−1^ *s*^−1^ can be taken as an upper limit of this important transport parameter, and the high value makes MAPbI_3_ a very promising material for solar cell applications. Finally, we note that the complexity of hybrid organic-inorganic structure of MAPbI_3_ as well as other perovskite matrerials may well lead to coexistence of various scattering mechanisms. In general, the dominant scattering mechanism that limits carrier mobility may be revealed by the calculated mobility value combined with other information such as its temperature dependence, as reported here.

## Methods

### DFT calculations

First principle calculations were performed via density functional theory (DFT) using the *Vienna Ab initio Simulation Package* (VASP)^65^. The exchange-correlation is treated at the generalized gradient approximation (GGA) level with the PBE method, as well as at the hybrid functional level with the HSE method^66^. In particular, HSE method gives the correct band gap. Projector augmented wave method is used to expand the electronic wave functions. The cutoff kinetic energy is set to 400 eV. A 3 × 3 × 3 Monkhorst-Pack k-mesh is used to integrate the first Brillouin zone. To optimize the supercell for tetragonal MAPbI_3_, internal positions of atoms and lattice parameters are relaxed until the residual force and total energy difference between the self-consistent steps are less than 0.01 *eV*/*Å* and 10^−6^ *eV*, respectively. When calculating the deformation potential under strain, only internal positions of atoms are relaxed. In our calculations, all structures are optimized with optb88-vdW functional^49^ to account for the van der Waals forces. Because there are heavy elements in the supercell, spin-orbit coupling effects are taken into account during the calculation of electronic properties^49^.

### Deformation potential scattering

The mobility limited by deformation potential scattering is calculated by the following equation^28^,^29^,^33^:


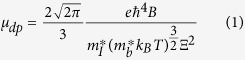


where 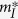 is the conductivity effective mass and 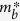 is the density of states effective mass, *B* is the bulk modulus 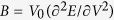, *k*_*B*_ is the Boltzmann constant, *ħ* is the reduced Plank’s constant, *e* the elementary charge and *T* the temperature. 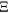 is the deformation potential coefficient that describes the change in energy of the band edge under elastic deformation, 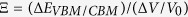. The mobility projected on three axis is calculated by^67^:


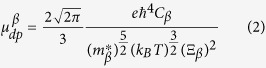


where *β* = *x, y, z. C*_*β*_ is the elastic modulus along direction *β*, and is derived from 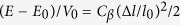, here *E* and *E*_0_ are total energies of systems under strain and equilibrium, respectively. *V*_0_ is the lattice volume at equilibrium. 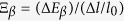 represents the deformation potential coefficient along the transport path, where Δ*E*_*β*_ is the energy variation of band edges owing to the compression and dilatation along the direction *β, l*_0_ is the equilibrium lattice constant in the transport direction *β* and Δ*l* is the variation of *l*_0_.

### Piezoelectric scattering

The piezoelectric scattering gives the mobility via the following expression^40^,^41^,^53^,^68^:


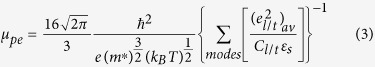


where 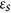 is the static dielectric constant, 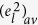 and 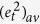 are spherical averages of the square of piezoelectric stress constants for longitudinal and transverse waves, respectively. *C*_*l*_ and *C*_*t*_ are the spherical elastic constant for the longitudinal and transverse waves, respectively. The summation is taken over all modes (longitudinal and transverse waves) for acoustic phonons.

## Additional Information

**How to cite this article:** Lu, Y.-B. *et al*. Piezoelectric scattering limited mobility of hybrid organic-inorganic perovskites CH_3_NH_3_PbI_3_. *Sci. Rep.* **7**, 41860; doi: 10.1038/srep41860 (2017).

**Publisher's note:** Springer Nature remains neutral with regard to jurisdictional claims in published maps and institutional affiliations.

## Supplementary Material

Supplementary Materials

## Figures and Tables

**Table 1:** Calculated mobilities via formula described by the deformation potential scattering mechanism and corresponding parameters for MAPbI_3_.

Carrier	Electrons	Holes
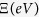	0.764	2.319
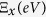	0.467	1.657
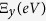	0.707	1.471
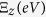	1.368	3.900
*B*(*eV*/*Å*^3^)	0.110	
*C*_*x*_(*eV*/*Å*^3^)	0.173	
*C*_*y*_(*eV*/*Å*^3^)	0.213	
*C*_*z*_(*eV*/*Å*^3^)	0.294	
*μ*(*cm*^2^ *V*^−1^ *s*^−1^)	0.303 × 10^6^	0.193 × 10^5^
*μ*_*x*_(*cm*^2^ *V*^−1^ *s*^−1^)	0.838 × 10^6^	0.520 × 10^5^
*μ*_*y*_(*cm*^2^ *V*^−1^ *s*^−1^)	0.379 × 10^6^	0.789 × 10^5^
*μ*_*z*_(*cm*^2^ *V*^−1^ *s*^−1^)	0.636 × 10^6^	0.247 × 10^5^

**Table 2:** Calculated mobilities limited by piezoelectric scattering mechanism and the corresponding parameters for MAPbI_3_.

Parameters	Values	Results	Values
*C*_11_(*GPa*)	20.100	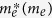	0.132
*C*_12_(*GPa*)	14.600	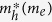	0.161
*C*_13_(*GPa*)	6.800	*C*_*l*_(*GPa*)	17.234
*C*_33_(*GPa*)	17.900	*C*_*t*_(*GPa*)	3.567
*C*_44_(*GPa*)	1.600	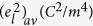	0.006
*C*_66_(*GPa*)	9.200	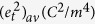	0.026
*e*_13_(*C*/*m*^2^)	−0.465	*μ*_*e*_(*cm*^2^ *V*^−1^ *s*^−1^)	11294
*e*_15_(*C*/*m*^2^)	0.066	*μ*_*h*_(*cm*^2^ *V*^−1^ *s*^−1^)	8368
*e*_33_(*C*/*m*^2^)	0.170		

The elastic constants tensors are employed from ref. 54 directly.
