# Supplementary material for: Piezoelectric scattering limited mobility of hybrid organic-inorganic perovskites CH3NH3PbI3
Source: Sci Rep. 2017 Feb 2;7:41860. doi: 10.1038/srep41860 (PMC5288793; doi:10.1038/srep41860)
Supplement: Supplementary Materials [file srep41860-s1.pdf]

# Piezoelectric scattering limited mobility of hybrid organic-inorganic perovskites $CH_3NH_3PbI_3$

Ying-Bo Lu[1,2,*], Xianghua Kong[2], Xiaobin Chen[2], David Cooke[2] and Hong Guo[2]

[1] School of Space Science and Physics, Shandong University, Weihai 264209, China
[2] Department of Physics, McGill University, Montreal, QC H3A 2T8, Canada

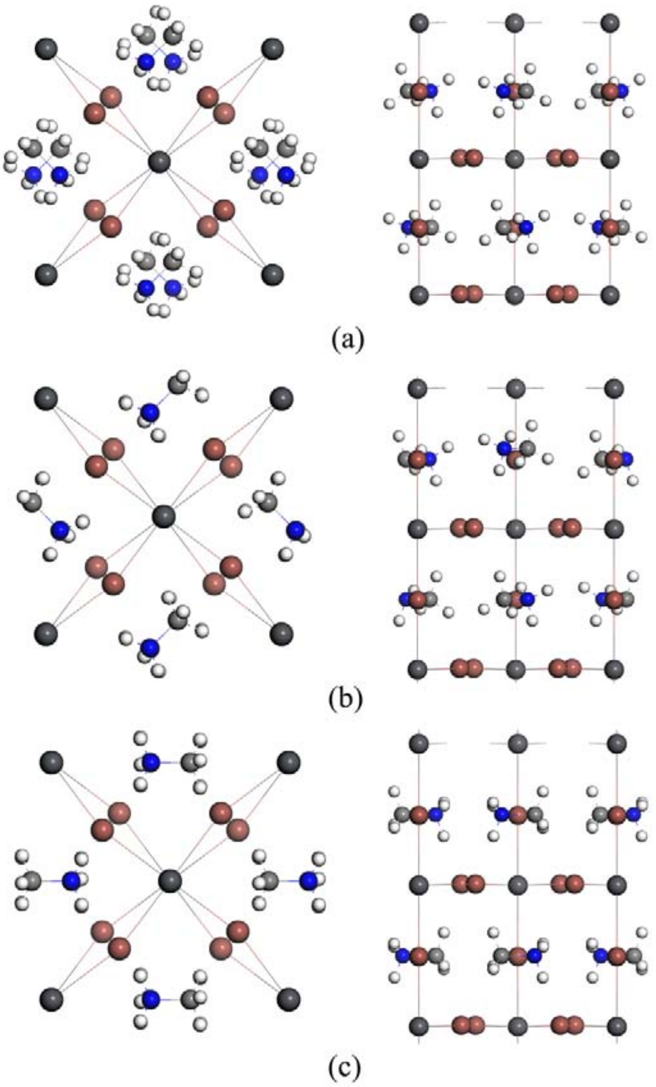


Figure S1. Three initial geometric structures of $MAPbI_3$ we employed to determine the most energetic stable configuration. These are top view (left side) and side view (right side) of configurations that containing (a) MA molecules along [110] direction but perpendicular with each other; (b) MA molecules along [110] direction but parallel with each other; (c) MA molecules along [100] direction with antiferro-electric arrangement.

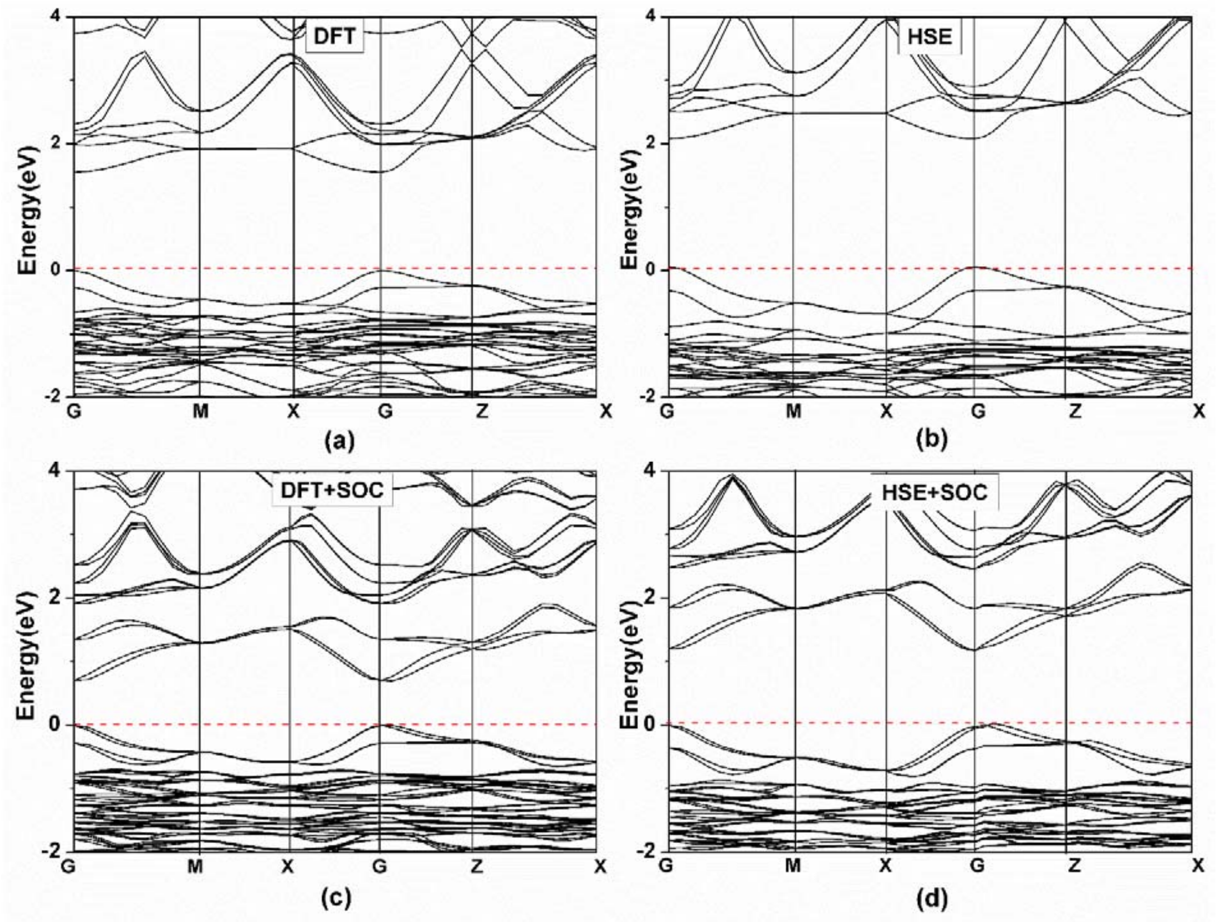


Figure S2. Bandstructures of tetragonal $MAPbI_3$ calculated via (a) DFT, (b) HSE, (c) DFT+SOC and (d) HSE+SOC functionals, respectively. The red dashed horizontal lines in these figures represent Fermi levels.

Table S1. The tabulate table of some reported mobilities for HOIPs materials to date.

| Compound | $\mu$(cm$^2$ V$^{-1}$s$^{-1}$) | | References |
|---|---|---|---|
| | $\mu_e$ | $\mu_h$ | |
| $MAPbI_3$ | 24.8±4.1 | 105±35 | Science , 2015 , 347(6225), 967 |
| $MAPbI_3$ | 8.1 | | Adv. Mater. 2014, *26*, 1584 |
| $MAPbI_3$ | 66 | | Inorg. Chem. 2013, 52, 9019 |
| $MAPbI_3$ | 800 | | Energy Environ. Sci., 2015, 8, 3700 |
| $MAPbI_3$ | 6.2 | | J. Phys. Chem. Lett. 2014, 5, 2189 |
| $MAPbI_3$ | 27(Tetragonal) | | J. Phys. Chem. Lett. 2015, 6, 4991 |
| | 150(orthorhombic) | | J. Phys. Chem. Lett. 2015, 6, 4991 |
| $MAPbI_3$ | 35(Tetragonal) | | Adv. Funct. Mater. 2015, 25, 6218 |
| | 150(orthorhombic) | | Adv. Funct. Mater. 2015, 25, 6218 |
| $MAPbI_3$ | 466-2046 (orthorhombic) | 140-614 (orthorhombic) | Nat. Commun. 2015, 6, 7383 |
| | 2577-11249 (orthorhombic) | 1060-4630 (orthorhombic) | |
| $MASn_{0.5}Pb_{0.5}I_3$ | 270 | | Inorg. Chem. 2013, 52, 9019 |
| $MAPbBr_3$ | 10.8 | | Nat. Commun. 2015, 6, 7961 |
| $MAPbBr_3$ | 115 | | Nature Reviews Materials 2016, 1, 15007 |
| $MAPbI_{3-x}Cl_x$ | 11.6 | | Adv. Mater. 2014, *26*, 1584 |
| $MASnI_3$ | 2320 | 322 | Inorg. Chem. 2013, 52, 9019 |
| $CsSnI_3$ | 536 | 520 | Inorg. Chem. 2013, 52, 9019 |
| $HC(NH_2)SnI_3$ | 103 | | Inorg. Chem. 2013, 52, 9019 |

Table S2. Carrier effective mass $m^*$ and band gaps $E_g$ of tetragonal $MAPbI_3$ calculated by various approaches. $e$ and $h$ in second row denote the carrier types of electron and hole, respectively. Subscripts of $x$,$y$ and $z$ for $m^*$ denote the carrier transport axis. DFT means using GGA functional for the exchange-correlation potential.

| Approaches | DFT | | DFT+SOC | | HSE | | HSE+SOC | |
|---|---|---|---|---|---|---|---|---|
| Carrier | *e* | *h* | *e* | *h* | *e* | *h* | *e* | *h* |
| $m_x$($m_e$) | 0.906 | 0.244 | 0.154 | 0.170 | 0.902 | 0.221 | 0.168 | 0.184 |
| $m_y$($m_e$) | 0.926 | 0.227 | 0.165 | 0.172 | 0.910 | 0.207 | 0.177 | 0.179 |
| $m_z$($m_e$) | 0.072 | 0.205 | 0.090 | 0.143 | 0.071 | 0.188 | 0.108 | 0.166 |
| $E_g$ (eV) | 1.552 | | 0.698 | | 2.024 | | 1.200 | |

Table S3. Angles between I-Pb-I bonds denoting distortions of $PbI_6$ octahedrons and rotation angles of MA molecules when $MAPbI_3$ is subjected to external strain along *x*-axis and *z*-axis, respectively. Symbols of angle $\boldsymbol{\alpha}$, $\boldsymbol{\beta}$, $\boldsymbol{\gamma}$ and $\boldsymbol{\delta}$ are illustrated in Figure F1, where former three angles represent in-plane tilting angles and the fourth one denotes the out-of-plane tilting angle, respectively. $\boldsymbol{\theta}$ is the rotation angle of MA molecule, where $\theta_z$ and $\theta_y$ are notations for angles along *z* axis and *y* axis, respectively. The positive and negative strains denote the tensile and compressive strains, respectively.

| Strain | *x*-axis | | | | | | *z*-axis | | | | | |
|---|---|---|---|---|---|---|---|---|---|---|---|---|
| | $\alpha$ | $\beta$ | $\gamma$ | $\delta$ | $\theta_z$ | $\theta_y$ | $\alpha$ | $\beta$ | $\gamma$ | $\delta$ | $\theta_z$ | $\theta_y$ |
| -0.030 | 93.066 | 176.791 | 174.477 | 176.865 | 33.707 | 44.752 | 91.450 | 176.089 | 173.365 | 175.665 | 33.067 | 50.720 |
| -0.025 | 92.77 | 176.601 | 174.243 | 176.602 | 33.735 | 44.390 | 91.450 | 176.075 | 173.394 | 175.760 | 33.176 | 50.253 |
| -0.020 | 92.554 | 176.381 | 173.939 | 176.334 | 33.707 | 44.088 | 91.449 | 176.062 | 173.410 | 175.843 | 33.176 | 50.253 |
| -0.015 | 92.301 | 176.310 | 173.810 | 176.265 | 33.625 | 43.938 | 91.448 | 176.007 | 173.399 | 175.868 | 33.300 | 49.645 |
| -0.010 | 92.001 | 176.186 | 173.691 | 176.246 | 33.571 | 43.787 | 91.450 | 175.961 | 173.386 | 175.867 | 33.408 | 49.202 |
| -0.005 | 91.735 | 176.041 | 173.515 | 176.109 | 33.585 | 43.757 | 91.451 | 175.945 | 173.382 | 175.887 | 33.449 | 48.979 |
| 0.000 | 91.448 | 175.918 | 173.365 | 175.868 | 33.517 | 43.547 | 91.448 | 175.918 | 173.365 | 175.868 | 33.517 | 48.754 |
| +0.005 | 91.198 | 175.937 | 173.354 | 175.921 | 33.490 | 43.456 | 91.451 | 175.920 | 173.393 | 176.027 | 33.585 | 48.553 |
| +0.010 | 90.903 | 175.900 | 173.319 | 176.002 | 33.368 | 43.337 | 91.451 | 175.891 | 173.416 | 176.140 | 33.653 | 48.326 |
| +0.015 | 90.601 | 175.808 | 173.256 | 176.093 | 33.274 | 43.248 | 91.448 | 175.828 | 173.423 | 176.259 | 33.734 | 47.854 |
| +0.020 | 90.325 | 175.702 | 173.150 | 176.170 | 33.261 | 43.127 | 91.449 | 175.710 | 173.387 | 176.338 | 33.843 | 47.390 |
| +0.025 | 90.054 | 175.672 | 173.116 | 176.138 | 33.167 | 43.008 | 91.458 | 175.651 | 173.401 | 176.420 | 33.924 | 46.948 |
| +0.030 | 89.790 | 175.577 | 173.014 | 176.197 | 33.209 | 42.796 | 91.459 | 175.482 | 173.328 | 176.468 | 33.992 | 46.697 |
